# APPLICATION OF CLASSICAL AND MACHINE LEARNING MODELS ON OASIS ‐ 2 DATA

**DOI:** 10.1002/alz.089953

**Published:** 2025-01-09

**Authors:** Rümeysa Rana Arslan

**Affiliations:** ^1^ Middle East Technical University, Ankara, Ankara Turkey

## Abstract

**Background:**

Alzheimer’s disease (AD) is a significant global health issue that affects both individuals and society for older adults. Early detection is essential in Alzheimer’s disease, as there is no definite treatment, and prevention is possible through early detection. In the studies of Alzheimer's disease, different approaches are used to understand the characteristics of the disease, including imaging methods such as MRI scans, biomarkers, and quantitative data from MRI scans. With these methods, the cognitive status of individuals could be determined. It is essential to detect the features affecting Alzheimer’s disease and detect the disease early and automate the detection and prediction by using modeling tools and by examining the datasets.

**Method:**

The symptoms of the disease can be observed over time, making the structure of the study longitudinal. Classical statistical models and machine learning algorithms can be used to analyze these datasets. The OASIS – 2 dataset is employed to find the features affecting dementia status and compare models' performances. The classical mixed models, their extended versions, and hybrid models, Boruta, GEE, GLMM, HGLM, GLMMLasso, GPBoost, GLMMTree, and HRF, are used to analyze the longitudinal dataset with a small sample size and binary outcome. Model performances are measured using accuracy, F1 score, sensitivity, and specificity, and the results are interpreted using the odds ratio.

**Result:**

GPBoost learns and classifies the dementia status well but overfits due to the small sample size in the dataset, and tree‐based algorithms are efficient in predicting the dementia status when a new subject enters the study.

**Conclusion:**

The common significant variables to address the dementia status of individuals are MMSE, nWBV, education, gender, and age. The results also indicate that the increase in MMSE score means the patient is less likely to be demented; the increase in normalized whole brain volume indicates a less probability to be demented; increase in socioeconomic status results in the patient having less probability to be demented; males are more likely to be demented; age has a negative impact on being demented; the increase in education years results in the patient having less probability to be demented.

